# The Use of Head-Mounted Display Systems for Upper Limb Kinematic Analysis in Post-Stroke Patients: A Perspective Review on Benefits, Challenges and Other Solutions

**DOI:** 10.3390/bioengineering11060538

**Published:** 2024-05-24

**Authors:** Paolo De Pasquale, Mirjam Bonanno, Sepehr Mojdehdehbaher, Angelo Quartarone, Rocco Salvatore Calabrò

**Affiliations:** 1 IRCCS Centro Neurolesi Bonino-Pulejo, Cda Casazza, SS 113, 98124 Messina, Italy; paolo.depasquale@irccsme.it (P.D.P.); angelo.quartarone@irccsme.it (A.Q.); roccos.calabro@irccsme.it (R.S.C.); 2 Department of Mathematics, Computer Science, Physics and Earth Sciences (MIFT), University of Messina, Viale Ferdinando Stagno d’Alcontres, 31, 98166 Messina, Italy; sepehrbaher@gmail.com

**Keywords:** motion capture, head-mounted display, virtual reality, upper limb kinematics, post-stroke, neurorehabilitation

## Abstract

In recent years, there has been a notable increase in the clinical adoption of instrumental upper limb kinematic assessment. This trend aligns with the rising prevalence of cerebrovascular impairments, one of the most prevalent neurological disorders. Indeed, there is a growing need for more objective outcomes to facilitate tailored rehabilitation interventions following stroke. Emerging technologies, like head-mounted virtual reality (HMD-VR) platforms, have responded to this demand by integrating diverse tracking methodologies. Specifically, HMD-VR technology enables the comprehensive tracking of body posture, encompassing hand position and gesture, facilitated either through specific tracker placements or via integrated cameras coupled with sophisticated computer graphics algorithms embedded within the helmet. This review aims to present the state-of-the-art applications of HMD-VR platforms for kinematic analysis of the upper limb in post-stroke patients, comparing them with conventional tracking systems. Additionally, we address the potential benefits and challenges associated with these platforms. These systems might represent a promising avenue for safe, cost-effective, and portable objective motor assessment within the field of neurorehabilitation, although other systems, including robots, should be taken into consideration.

## 1. Introduction

Human upper limb tasks require fine-tuned coordination of multiple joints and muscles for interaction with the surrounding environment. Upper limb functions are often impaired after a brain injury [1]. In 2017, it was estimated that approximately 1.12 million people in Europe were affected by cerebrovascular disorders, due to ischemic or haemorrhagic events [2]. Post-stroke patients can manifest altered motor patterns in the contralesional upper and lower limbs, due to paralysis and/or spasticity [3]. In detail, infarctions of the middle cerebral artery affecting the primary motor cortex and the integrity of the corticospinal tract have been associated with upper limb movement deficits (including weakness, decreased inter-joint coordination, and diminished finger dexterity) [3,4]. Over the last few decades, upper limb kinematic assessment has grown in popularity in a clinical context. Indeed, it has been increasingly used as a more objective outcome, especially in stroke patients. During robotic-assisted therapy, the use of kinematic assessment is fundamental to monitor the patient’s progress and to tailor the rehabilitation process to their needs. It is noteworthy that some robotic devices can perform a complete kinematic assessment automatically, as suggested by Bonanno and Calabrò [5]. In detail, a new category of robotic devices, known as collaborative robots, is growing in popularity in the field of rehabilitation. Due to their inherent safety and reliability, collaborative robots are increasingly being explored in healthcare applications, including robotic rehabilitation and kinematic assessment, where they can provide physical interaction with patients driven by actuation systems [6].

The advantage of upper limb instrumental kinematic measurements, compared to standard clinical assessments (e.g., Fugl-Meyer, Box and Block, 9-Hole Peg Test), is that different aspects of motion can be tracked more objectively and continuously [4]. However, the diversity and heterogeneity of kinematic assessment techniques, instrumentation, and selected outcomes expanded along with this developing area, make it challenging to interpret results. Moreover, the upper limb tasks comprise a wide set of activities, including “non-contact” movements such as gestures, or “contact” movements such as grasping activities [7]. This assessment can be performed in dedicated environments, like camera-based motion laboratories, or using robot-based measurement systems [5,8,9].

In the literature, the most used motion capture (MoCap) systems are based on optoelectronic technology, which is often considered as the “gold standard” because it is the most accurate and reliable when compared to other MoCap technologies [9]. Specifically, optoelectronic systems detect light and estimate the 3D position of a marker by triangulation from multiple cameras. They involve two main categories of markers: passive and active [9]. The passive marker system uses markers that reflect infrared (IR) light to the cameras, e.g., the VICON (Oxford, UK) or Optitrack (Corvallis, OR, USA) systems, whereas active marker systems contain a source of light for the sensors (often IR), provided that they are powered. To reconstruct the motion of a skeletal segment, reflective skin markers are placed on anatomical landmarks, and kinematics are derived from the marker position by adapting them to a human body model [10,11]. Despite the accuracy and reliability of optoelectronic systems, the widespread implementation of such devices as large-scale screening tools is impeded by their costs. Consequently, their usage is confined only to specialised clinical laboratory settings [10]. Over the past decade, the advent of novel technologies for motion analysis utilising wearable inertial sensors has paved the way for new approaches in clinical practice for the delivery of patient-centred rehabilitative interventions [8,12]. Inertial measurement units (IMUs) are small devices that integrate different multiaxial sensors, like accelerometers, gyroscopes, and magnetometers, to detect movements. These systems are particularly popular since they have several advantages, being low-cost, wearable, easy-to-use, settings which allow continuous monitoring of movement both in the clinic and at home [8]. IMUs collect raw data from each integrated sensor through on-board sensor-fusion algorithms, based on Kalman filters [13], in order to obtain different motion parameters. Generally, these systems allow the recording of kinematics, providing data on angular acceleration, velocity, and spatial orientation, from different body parts, including upper limbs [8,10]. However, the main disadvantage is that IMUs are less accurate and reliable than optoelectronic systems. In fact, IMUs estimate the orientation of body segments which may be likely to cause drifting due to the integration of noisy measurements [10]. To overcome this issue, there are numerous strategies allowing the corrections of potential bias in the motion analysis. Another MoCap method consists of the image analysis captured by one or more cameras through computer vision algorithms, which extracts information to describe movement. These tracking systems exploit machine learning methodologies, facilitating the recognition of human body movements, hand gestures, and expression of the face [14].

### Virtual Reality Head-Mounted Display (HMD-VR) Platforms

On the other hand, novel technologies have recently caught on in clinical settings, like virtual reality (VR) platforms which integrate MoCap systems. Virtual reality head-mounted displays (HMD-VR) technology offers various possibilities to track body posture. These include specific marker positions, or a camera integrated in the helmet combined with computer graphic algorithms to estimate hand position and gestures. VR employs real-time rendering to generate immersive visual experiences, constantly updating images based on the users’ movements within the virtual space. Another method to provide virtual feedback is augmented reality, which combines the real world and computer-generated 3D content. Augmented reality adjusts the ongoing perception of a real-world environment, while VR entirely substitutes the user’s real-world environment with a simulated one. These systems can incorporate visual, auditory, and haptic feedback (i.e., hand controller vibration) to enhance the sense of immersion. Visual and auditory feedback are widely integrated in VR setups, but haptic technology remains neglected, often limited to adjustable vibrating motors found in handheld controller or wearable tracker devices. These haptic systems can modulate intensity, frequency, and duration to enhance user experience [15]. Other devices can be used and integrated in HMD-VR platforms to provide a wider range of haptic vibration feedback, such as haptic gloves, vests, and whole-body suits [16,17], or haptic force magnitude feedback [18]. However, these devices can significantly increase the overall price of the HMD-VR system and are often prototypes and not available on the market yet. Thanks to the gaming world, the HMD-VR has made great strides in recent years in terms of system usability and reliability of movement estimation. With positional tracking, users can navigate within the virtual environment along the X, Y, and Z axes, enabling not only head rotation but also horizontal and vertical movement. In addition, hand controllers, as well as additional trackable devices, are equipped with sensors to monitor the users’ interactions within a virtual space. The possibility of easily creating controlled virtual experimental environments integrating different types of sensors within such platforms, through opensource development libraries, has opened the way for complex rehabilitation applications [19,20].

Moreover, HMD-VR are easy-to-use and low-cost systems, which allow users to perform rehabilitation treatments for both inpatients and outpatients [21]. In the neurorehabilitation context, the HMD-VR platforms were mostly used to train upper limb functions in post-stroke patients. According to recent studies [22,23], these systems are promising tools used to improve upper limb functional outcomes and daily living activities in chronic post-stroke patients. HMD-VR platforms offer the possibility of performing task-oriented movements with high degrees of realism, which could enhance gesture relevance [21,24]. According to some studies, post-stroke patients reported high levels of satisfaction, engagement, and motivation during rehabilitation treatment with VR as well as HMD-VR, denoting an overall acceptance of the device. Despite their promising role in the rehabilitation context though, it is still not clear if they are reliable and advantageous systems when compared to the classical MoCap.

In this review, we aim to report the state of the art of HMD-VR used for the kinematic analysis of upper limbs in post-stroke patients, offering a comparison between the conventional MoCap and HMD-VR. Moreover, the emerging role of robots and collaborative robots with virtual and augmentative reality has also been investigated.

Given the narrative and perspective nature of this review, we have analysed the most relevant studies in the English language (such as studies with different designs, focusing on the kinematic evaluation of upper limb motor tasks in post-stroke patients, using HMD-VR compared with conventional MoCap). Finally, each article has been assessed by the authors considering its title, abstract, text, and scientific validity.

## 2. Kinematics of Upper Limb Movements in Post-Stroke Patients

Differently from lower limbs, upper limbs have many degrees of freedom, allowing subjects to perform fine movements of reaching and grasping for daily life activities (ADL). Hand and arm movements constantly influence human ADL and sensory functions, providing a constant interaction with the environment [7,25]. In terms of kinematics, the upper limbs are an open kinematic chain starting in the sternoclavicular joint and ending in finger joints [26]. Due to the specific structure of human proximal joints, only rotational motions in these joints are possible [26]. Upper limb movements also involve object manipulation, which entails controlling finger forces to execute specific grasp types. For instance, when reaching for an object, the hand adjusts to its location and shapes the fingers to grasp it securely [27,28]. During planar reaching tasks, where the aim is to reach a target at a set distance by moving the arm and/or forearm, healthy individuals swiftly move towards the target. If the target is far away and the task takes longer, they may make a secondary corrective movement based on sensory feedback (like visual cues) to adjust their trajectory. Healthy individuals not only achieve consistent accuracy in reaching the target, but they also adjust their movement speed to maintain a fairly constant duration [25].

Upper limb reaching or hand and arm grasping actions may be impaired in post-stroke patients [28,29]. These patients can manifest weakness related to the inability to activate specific upper limb muscles or segments with uncoordinated inter-joint movements [28,29]. This abnormality is caused by an irregular flexor synergy during reaching, resulting in reduced finger dexterity for grasping and an increase in trunk movements as compensation. Pathological flexor synergy entails abnormal muscle activation, resulting in an uncoordinated contraction of elbow flexors and shoulder abductors. This phenomenon becomes apparent during upper limb tasks necessitating fine motor control and finger manipulation [25,26,28,29]. After a stroke, patients typically have slower hand movements and may take longer to complete grasping actions. Their grip openings tend to be wider, and they may move their hand along a longer path while performing grasping tasks. Furthermore, they often experience reduced grip strength while grasping and lifting objects [26,30]. Stroke survivors employ various compensatory strategies to improve grasping, including narrowing finger spread and adjusting finger and hand joints. These adaptations aim to enhance their ability to grasp objects effectively after stroke [4,29].

In this sense, functional assessment of the upper limbs is fundamental to plan rehabilitation and treatment of post-stroke patients according to their needs. By or large, physiotherapists are used to administering clinical scales and tests to assess upper limb functionality. For example, the Fugl-Meyer Assessment (FMA) scale is often administered to patients with stroke to evaluate functional abilities of the shoulder, elbow, wrist, and hand [7]. Despite its large use in clinical practice, this scale does not allow for sufficiently discriminating physiological and the above-mentioned pathological movement behaviour. To this aim, new emerging technologies are growing in the field of neurorehabilitation. In recent years, kinematic analysis has increasingly been used to objectively assess upper limb motor function after stroke and evaluate therapy effectiveness [4,31]. Unlike gait analysis, upper limb kinematic assessment does not have specific evaluation protocols, which makes the kinematic assessment more difficult. Previous studies have used different tasks and MoCap systems to illustrate the kinematic features of upper limb motor impairments following stroke (see Table 1).

These tasks often simulate ADL such as drinking, carrying, reach-to-grasp motion, or playing games. The Second Stroke Recovery and Rehabilitation Roundtable (SRRR2) [32] suggested that post-stroke patients with moderate to severe upper limb impairments may benefit from simpler point-to-point tasks, also known as two-dimensional (2D) tasks [25,31]. Examples of these tasks include Hand-to-Mouth (HTM) [33], Finger-to-Nose [34], or LIGHT tasks [35]. This recommendation aimed to make kinematic analysis more accessible and valuable for these patients. For example, Huang et al. [36] evaluated the HTM in post-stroke patients. They analysed kinematic variables such as movement smoothness, movement velocity, movement trajectories, and the range of movement (RoM) of trunk and upper limb joints, using a 3D MoCap system. Similarly, Shwartz et al. [4] conducted a kinematic motion analysis of the upper limbs in post-stroke patients. They examined parameters including trunk movements (e.g., displacement), shoulder, forearm, and wrist movements, as well as movement time, peak velocity, the number of velocity peaks (NVP), and spectral arc length (SPARC).

Choi et al. [37] measured upper limb parameters in the sagittal, coronal, and transverse planes. Indeed, the authors also considered the thoracic angles and RoM in tilt obliquity, and rotation at the four points between each phase. Also, angle deviations for each joint angle were calculated during the task to estimate the degree of movement deviation, comparing them with healthy controls. On the other hand, Guzik-Kopyto et al. [38] identified 16 parameters, encompassing maximum joint angles and motion ranges of the joints, spinal kinematics to detect compensatory movements, and the velocity of movements to assess upper limb physical efficiency and capability.

Aprile et al. [39] assessed the kinematics of drinking activity in post-stroke patients, examining parameters like arm elongation, trunk forward inclination, and trunk axial rotation to understand their impact on reaching movements. In addition, the authors calculated the RoM of the elbow during the reaching tasks, and measured mouth forward displacement during specific phases to determine trunk inclination.

Kinematic analysis is vital in post-stroke patients for quantitatively detecting movement alterations, including compensations and pathological synergies. It also enables the monitoring of progress following rehabilitation interventions. In addition, it could influence treatment decisions and maximise recovery. This may also improve the confidence of patients in the efficacy of the interventions during the chronic stage.

## 3. Technologies for Motion Capture: Optoelectronic, IMUs, and Vision-Based Motion Tracking and Other Solutions

In this paragraph, we aim to describe the most common MoCap systems used for kinematic motion analysis. We have explored different types: optoelectronic, IMU, and vision-based motion tracking, as well as robotic devices and HMD-VR, pointing out their technical features and differences (see Table 2).

**Figure 1:**
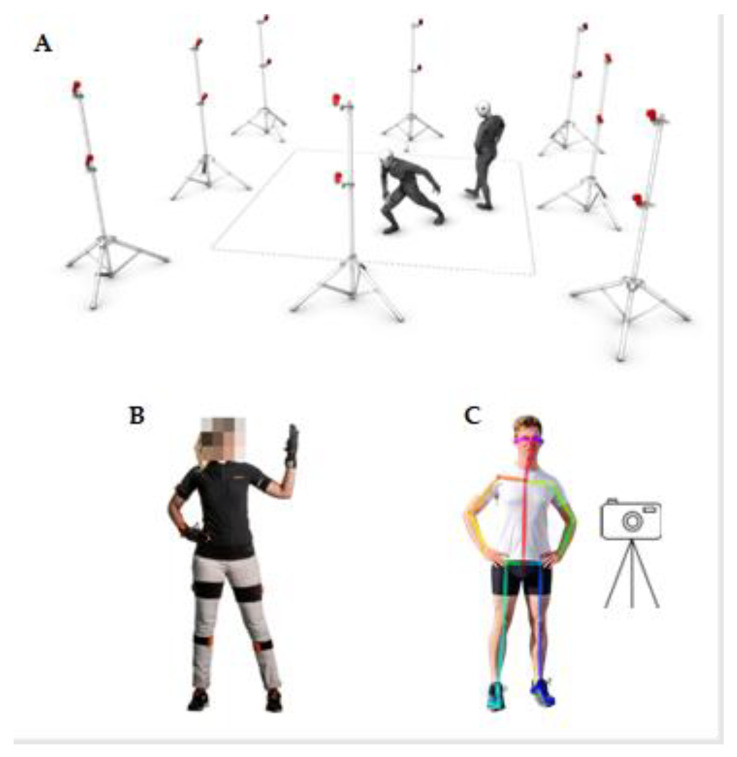
Conventional MoCap systems used for kinematic analysis of upper limb. (**A**) An example of a commercial optoelectronic MoCap system setup (Optitrack Flex 13 Motion Capture Camera [40]), which consists of several IR cameras where subjects are instrumented with reflective markers; (**B**) one of the subjects, who is wearing one of the commercially available IMU Mocap systems (Xsens Awinda [41]); (**C**) VBMA key points detection using an OpenPose pose estimation algorithm through a camera [42].

Optoelectronic devices represent a valid instrument for upper limb functional assessment providing a valid tool for 3D kinematic motion analysis [9,35]. These systems use light to estimate the 3D position of a marker by triangulation from multiple cameras, and they can use active and passive markers [9,10]. The latter are covered by photo-reflective materials that reflect the infrared light, while active markers emit IR light (see Figure 1A).

The 3D kinematic analysis of the upper limbs using optoelectronic devices can vary significantly in terms of marker sets, kinematic models, analysed functional movements, and reported kinematic outcomes. By or large, two sets of markers are used: anatomical or cluster marker sets. The former is placed in correspondence with body landmarks to build kinematics models, as anatomical landmarks are essential to define the local coordinate systems of a body segment (e.g., scapula, humerus, and thorax) in static conditions. On the other hand, cluster marker sets are placed in different body segments. These markers are calibrated to correspond to specific anatomical landmarks, facilitating accurate tracking of movement [43].

Motion recording systems based on inertial sensors allow the estimation of movement and orientation of specific body segments [8] (see Figure 1B). These systems typically rely on data from integrated accelerometers, gyroscopes, and magnetometers within compact, lightweight devices attached to the targeted body region. Accelerometers register velocity alterations along the sensor’s three axes, enabling estimation of velocity and spatial displacement through integration processes. Gyroscopes measure angular velocity, enabling tracking of orientation changes, while magnetometers detect magnetic field strength and direction, providing absolute orientation relative to the Earth’s magnetic field [8,10].

Position estimation based on inertial systems is not a direct process; indeed, it begins with estimating velocity from acceleration and then proceeds to estimating position from velocity through a double integration process.

This technique is known as dead reckoning (path integration), a navigation method used to estimate the current position based on a previously determined one, utilising speed, elapsed time, and course direction [44].

Commercial systems may comprise a single sensor, like the BTS G-WALK (BTS Bioengineering S.p.A., Milan, Italy), or multiple sensors like Xsens Link system (Movella Technologies, Enschede, The Netherlands), contingent on application requirements. For example, a single sensor positioned at the sacral level, combined with mathematical models, is adequate for evaluating walking, running, and jumping performances [10].

Conversely, complex kinematic analyses, like upper limb reaching tasks that involve numerous degrees of freedom, require the integration of multiple sensors and more sophisticated mathematical models [45].

Sensor placement typically involves specialised suits or elastic bands tailored to the target segment. Inertial systems are considered feasible also in post-stroke patients, as demonstrated by Held et al. [46]. To collect kinematic data, these authors used an Xsens full-body motion capture suit. In particular, IMUs registered kinematic parameters of movement during clinical assessment, both at the hospital and in home settings. This aspect is extremely advantageous because IMUs systems allow for the quantification of kinematic analysis outside a laboratory environment [8,28].

However, when comparing IMUs to optoelectronic systems, they are considered less accurate. This is because they do not directly record position (unlike optoelectronic systems); instead, they estimate it from acceleration [47].

However, when comparing IMUs to optoelectronic systems, they are considered less accurate. Secondly, IMU systems notably suffer from drift accumulation which leads to gradual change or offset in their output readings over time. This drift can be caused by various factors, including temperature changes, aging of internal components, imperfections in manufacturing, and electronic noise. Thirdly, metal objects in the evaluation environment may cause electromagnetic interference and significant distortions. Furthermore, conducting precise analyses involving multiple joints often necessitates the use of several sensors. However, due to the finite resolution and dynamic range of accelerometers, measurements of extremely small or large accelerations may not be reliable. [10,47]. On the other hand, IMU systems, as outlined in Table 2, provide significant advantages. They offer large capture volume capabilities and are portable, lightweight, and wireless. Additionally, they are adaptable to various environments, including outdoor conditions [48,49]. They boast cost-effectiveness and minimal recording latency, facilitating real-time applications. This is why they are often used for recording in real-time situations. In post-stroke patients, Nie et al. [50] analysed wrist reaching movements, comparing optoelectronic and IMU devices. The authors found that the mean error between the two systems was 0.09 ± 1.81 cm.

Moreover, another branch of MoCap is revolutionising the field of movement analysis, such as vision-based motion analysis systems (VBMA). VBMA is a type of markerless motion capture, which extracts information from subsequential images to describe movement [10]. Generally, this type of MoCap uses cameras with high resolution to ensure high accuracy. Hence, these tracking systems incorporate cameras or camera arrays to facilitate the recording of human body movements. Subsequent application of computer vision and machine learning techniques enables the capture of gestures of the hands and expressions of the face [14].

Significant implementations of these technologies include software libraries like MediaPipe [51], MoveNet [52], and OpenPose [53] (see Figure 1C). These libraries empower developers to cover a wide area of the image and track multiple individuals simultaneously, with numerous points, reaching up to 500, and including finger and facial features. Technical evaluations indicate that fast movements or considerable distances from the camera can cause a notable decline in the accuracy of segment recognition. This often leads to incorrect delineation of skeletal nodes. A significant limitation arises from the inability of a single camera to accurately establish the third coordinate of points (along the *Z*-axis), which makes it impossible to determine the distance between the camera and the object. Nonetheless, these limitations may be less impactful in certain musculoskeletal rehabilitation exercises, particularly those involving simple movements along one or two axes. Alternative solutions such as camera arrays, specialised cameras equipped with depth sensors or stereo capabilities provide opportunities for capturing the necessary data on the positioning of body parts [14,54]. According to Faity et al. [55], the Kinect, a device for VBMA, was reliable in assessing trunk compensations, hand range of motion, movement time and mean velocity with a moderate to excellent reliability, in post-stroke. In contrast, this system did not show the same accuracy in estimating elbow and shoulder range of motion, time to peak velocity, and path length ratio.

### Other Technologies for Motion Capture: Focus on Collaborative Robots

Beyond conventional MoCap, other technologies can perform an objective and quantitative kinematic motion analysis, such as robotic devices. In fact, these technologies are commonly used for rehabilitation purposes, but they also can be translated to both analyse the patient’s limb trajectory and accurately register spatial–temporal parameters of movement, collecting a large amount of data. These devices may be characterised by an end-effector attached to a set of arms connected by proper joints. Thanks to kinematic calculation methods and integrated measurement devices, the position of the end-effector or the arms can be measured [5]. In particular, robots integrate measurement devices, called encoders, which record joints angular deviations through optical, mechanical, or magnetic technology. These devices provide a reliable and objective motion analysis, also during rehabilitation training. For example, some authors [56,57,58] evaluated the range of joint upper limb movements, movement velocity, accuracy, and smoothness in active training, using robotic devices (i.e., Armeo Power, Armeo Spring).

Moreover, within rehabilitation, robotic devices can play pivotal role in upholding and overseeing the quality of users’ movements, which is essential for customised treatment effectiveness. Consequently, robot-assisted therapy is gaining attention as a method for addressing motor function issues, particularly in the upper limbs, facilitating repetitive, high-intensity training while ensuring movement precision [59,60,61]. Incorporating robots can also diminish the necessity for continuous, hands-on supervision by therapists, as they can automate tasks and track the speed and accuracy of movements continuously [59,60,61]. Collaborative robots, called also cobots, are a small class of industrial robotic devices able to reduce human effort and minimise the risk of high-velocity impacts or injuries by collisions. In general, cobots can be differentiated from traditional robots by their flexible characteristics and safety features, which are provided by sensor-based control methodologies [62]. Additionally, these systems can use augmented reality, and may be customised for each patient.

Some authors [63,64] used cobots for both rehabilitation and motion evaluation purposes. In fact, all the data from the instrumented robot arm is available and the process can be monitored with accuracy. The system can also integrate decisions on increasing the difficulty of the training and its progress in order to motivate the patients. For example, Kyrkjebø et al. [63] used an industrial cobot (UR5e) as a rehabilitation tool for upper limb training in post-stroke patients. The authors found that this device is feasible, since the robot can be customised to execute a wide range of movements suitable for the treatment process. In addition, the feasibility of cobots is enhanced when it is combined with accurate force and torque measurements.

Reinkensmeyer et al. [64] used a lightweight exoskeleton (Pneu-Wrex) to train upper limbs in post-stroke subjects, allowing a wide RoM of the arm in a 3D space by incorporating pneumatic actuators to generate active forces. In particular, the robot constantly monitored the assistance the patient needed to achieve the current task, and then provided slightly less assistance than the estimated amount. In this way, post-stroke subjects were encouraged to make more effort, improving their motor skills. Rodrigues et al. [65] combined cobots with augmented reality.

This work proposes grouping a collaborative robot with one specific augmented reality equipment to create a rehabilitation system where some gamification levels might be added to provide a better and more motivating experience to patients.

To date, studies on robotic devices have mostly focused on helping patients with neurological disorders improve their motor skills and functional recovery. However, the potential role of robots, regarding cobots, in the growing field of motion analysis deserves further investigation.

## 4. Benefits and Challenges of Virtual Reality Head-Mounted Display Platforms

Given the remarkable diversity observed in literature regarding the technology used, the upper limb motor tasks performed, and the kinematic metrics analysed, specific recommendations have been developed. These recommendations aim to establish standardised methods for assessing upper limb kinematics after stroke [33]. However, in recent years, an increasing number of motion analysis technologies have become commercially available, such as HMD-VR, which have different characteristics and features that have not yet been fully investigated in the field of rehabilitation [19]. In this paragraph, we have shed some light on the benefits and challenges of the different types of HMD-VR platforms compared with conventional marker-based MoCap systems, since they are considered as the “gold standard”.

HMD-VR platforms, which are portable and low-cost, serve dual purposes: motor training and objective kinematic analysis of the upper limbs (see Table 2). HMD-VR platforms integrate validated technologies such as optoelectronic, IMUs, and VBMA for motion tracking to estimate the user’s position in virtual space [20]. Obtaining a good estimate of the user’s position is necessary to move and interact within VR environments. In HMD-VR platforms, the environment is displayed within the helmet, and the view of the physical world is completely obscured. Typically, these systems use head and hand position tracking for most applications. More complex applications may also require tracking of other body parts using multiple sensors or integrating data within biomechanical models [66]. In detail, HMD-VR incorporates various technical features to enhance the user’s experience. These include high-resolution displays for detailed visuals, wide fields of view for immersion, and high refresh rates for smooth motion. Tracking technology monitors the user’s head movements and position, while comfort features like adjustable straps and lightweight designs ensure prolonged use. Built-in audio systems or support for external headphones provide spatialised audio, and connectivity options enable interaction with external devices. Additionally, some HMDs come with handheld controllers and additional wearable trackers to interact with the virtual environment and record kinematics. Adjustable straps positioned on body segments enable recording of paretic limb motion without requiring the user to hold the controllers. These features collectively contribute to the performance, comfort, and immersion provided by HMDs in VR applications [67]. Moreover, these systems should possess adaptability for diverse settings and pathologies, as well as scalability features. Specifically, HMD-VR systems exhibit adaptability, providing portability, integration with other technologies (such as sensors and computers), interactivity via hand gestures, voice commands, and motion controllers, as well as comfort and ergonomic features (including adjustable headbands, cushioned padding, and lightweight materials). It is noteworthy that the adaptability of HMD-VR as a treatment device for other neurological patients has already been already demonstrated for use in treating Parkinson’s disease [68] and in traumatic brain injury [69].

In addition, HMD-VRs offer scalability features, related to the following: (i) HMD-VR systems (e.g., these systems allow switching from one HMD-VR to another); (ii) degrees of VR (e.g., visual quality and latency which depend on the type of feedback and the desired performance); (iii) numbers of collaborators (e.g., numbers of users connected simultaneously in the same VR environment). However, it is not a common practice nowadays to use and interact with multiple users within VR, probably due to a lack of appropriate haptic feedback; (iv) the number of recorded objects/joints/limbs is based on the type of technology (e.g., in HMD-VR outside-in, the number of recorded objects depends on the number controllers/trackers presented in the scene and integrated within the device; however, in HMD-VR inside-out tracking, additional controllers (marker-based) need integration, or complex mathematical models (markerless) must be employed, ensuring they remain within the camera’s field of view) [70].

A promising trajectory in motion analysis lies in the direction of a fully automated, non-intrusive method. Such an approach has the potential to represent a significant advancement for both research and practical applications within the domains of biomechanics and neurorehabilitation [19,21]. Nevertheless, most clinical studies have investigated the effects of the HMD-VR platform as a tool for rehabilitation intervention, due to its enriched and controlled environment [19,21]. Relatively little attention has been directed towards the potential utility of HMD-VR as kinematic measurement devices. Notably, only a handful of studies have examined the kinematic accuracy of HMD-VR in comparison to conventional MoCap systems [66,71], and even less research has investigated this issue in post-stroke patients [50,72].

**Figure 2:**
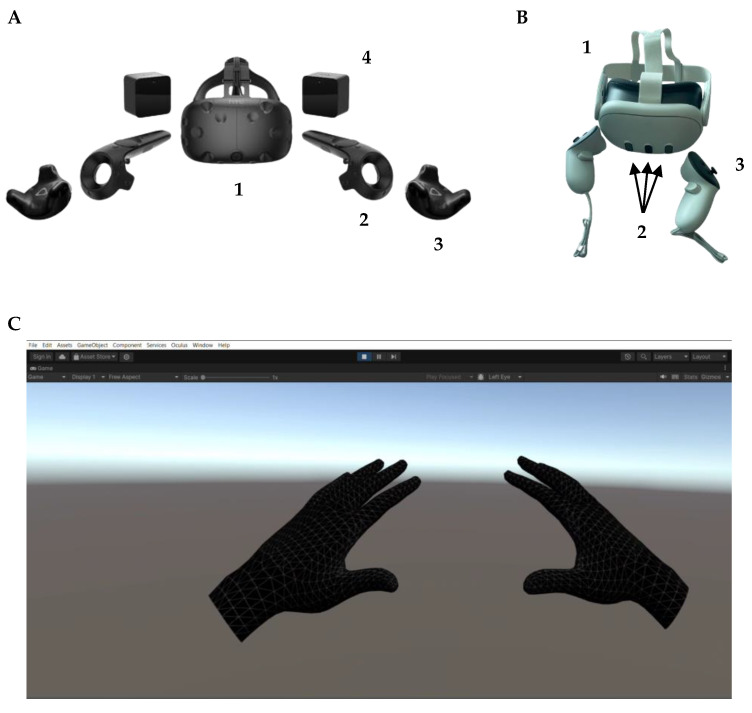
HMD-VR platforms. (**A**) An outside-in HMD-VR platform (HTC Vive, HTC Europe Co., Ltd., Slough, Berkshire, UK). This system consists of an HMD (1), 2 controllers (2), 2 trackers (3), 2 base stations or “lighthouses” (4). (**B**) An inside-out system (Meta Quest 3, Meta Technologies LLC, New York, NY, USA), which simply consists of an HDM (1) with integrated cameras (2) and 2 controllers (3). (**C**) Three-dimensional hand model representation developed with Unity [73] software (version 2023.2.1f1) and the device’s software development kit (Meta XR Core SDK, version 65.0) [74].

Nowadays, there are different methods that rely on different technologies to track the user’s position: outside-in (Figure 2A) and inside-out (Figure 2B) [10,66].

**Figure 3:**
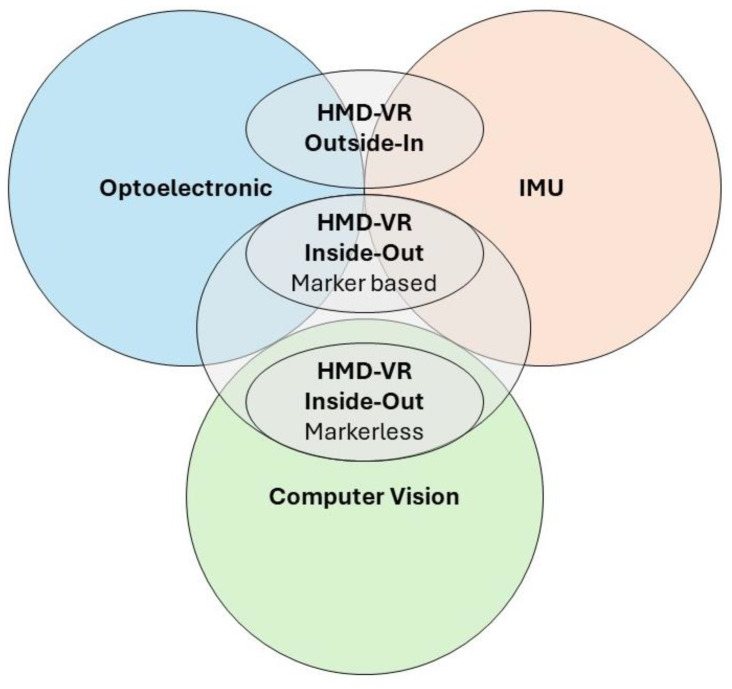
Tracking systems for HMD to estimate upper limb motion. Figure shows motion tracking technology exploited by HMDs to estimate upper limb (end-effector) position.

The first systems commercially available have outside-in technology; that is, they allow the position of the HMD (Figure 2A, 1) and handheld controllers and wearable trackers (Figure 2A, 2, 3) to be estimated through external cameras or sensors (Figure 2A, 4). These systems, such as the HTC Vive (HTC Europe Co., Ltd., Slough, Berkshire, UK) or the Oculus Rift (Oculus, Irvine, CA, USA), integrate information from external cameras with IMUs placed inside the wearable devices, allowing reliable and accurate estimation even in large working environments [24]. The IR light-sensitive cameras rely on optical technology to estimate the position of a given set of markers that is placed on the HMD or controller. Typically, two “lighthouse” station bases emit flashes from an IR LED array at a fixed frequency, along with vertical and horizontal lasers sweeping in both horizontal and vertical directions across the room. By analysing the sequence in which the photosensors on the HMD and controllers receive these laser sweeps, the position of the tracked devices can be determined [66]. Since the external base stations are placed in stationary locations, the estimated position of the tracked objects depends on the lighthouses. Indeed, a calibration procedure is required to set system components and achieve reliable and accurate measurements. The tracking volume depends on the type and placement of the base stations. Considerable tracking spaces can be achieved up to a maximum size of 7 m × 7 m. For object tracking, it is only necessary that the objects are within the tracking volume of the base stations. The data stream from the HMD-VR platforms reaches the PC through a cable. Graphics processing is then carried out by the PC, which is responsible for updating the virtual scene [75].

On the other hand, inside-out systems have been gaining popularity in recent years, since their features are more suitable in the field of gaming and entertainment than the outside-in systems. First, the main difference lies in the fact that in the inside-out systems, the camera is not located externally but physically inside the HMD (Figure 2B, 2) [75].

Two different types (i.e., marker-based, and markerless) of tracking objects modalities are possible within inside-out systems. In particular, when the application requires a controller to interact with the VR environment, the same technology of outside-in systems, based on IR and IMU, is used. However, in contrast to outside-in systems, inside-out systems do not rely on external cameras or base stations to detect controller position. Instead, in these cases, the IR LED in the controllers is detected by HMD cameras, which integrate this information with data from multiple IMUs placed on the controllers [76]. The inside-out systems also allow the user to interact with the VR environment using hands and without any markers [77]. A combination of computer vision algorithms, including visual-inertial mapping, place recognition, geometry reconstruction, and cameras placed on the HMD, can be employed to estimate the position and gesture of the hands [72]. Position and gestures are extrapolated from the recorded images through the integration of stereoscopic information obtained from the cameras and VBMA [14], as shown in Figure 2C. For example, in Figure 2C, hand pose and position, extrapolated from recorded images, are shown through a 3D model representation. This 3D model representation was developed with Unity (Unity Technologies, San Francisco, CA, USA) [73] software, which is one of the most popular VR environments development software. In addition, Unity software allows 2D and 3D VR environment developments on different platforms, like desktop, mobile, augmented, and virtual reality. To aid and facilitate multi-platform content development, different tools such as the software development kit (SDK) and specific open source software, like SteamVR (Valve Corporation, WA, USA) [78], provide a wide set of libraries.

Regarding the tracking volume, significant differences are evident among the different types of HMD-VR systems. In inside-out devices, the tracking volume is limited by the visual space of the cameras that are on the helmet. Therefore, the volume is dynamic and restricted when it is compared to outside-in systems. Objects can therefore be tracked if they are within the field of view of the cameras [79]. If hands are beyond the camera’s field of view, tracking becomes unfeasible, except in marker-based situations where position estimation is still achievable, albeit with less reliability and for a limited duration, solely relying on IMUs. (see Figure 3).

These systems allow untethered, i.e., unconstrained configuration [80]. All processing of the virtual scene and updating of object positions within the room as well as the viewpoint itself, are handled directly by the HMD. Alternatively, when high graphical or computational demands are present, this processing can be offloaded to a PC via cable. Given that, both HMD outside-in and inside-out present different characteristics and meeting points, which are all displayed in Table 3.

Considering the aforementioned aspects, HMD-VR platform and conventional MoCap systems differ for tracking performance accuracy and precision. Some authors have compared these technologies in both healthy and post-stroke patients (see Table 4).

For example, Nie et al. [50] evaluated the tracking accuracy (0.48 ± 1.58 cm) of the wrist during the reaching task in post-stroke optoelectronic, outside-in HMD-VR. On the other hand, some authors compared the tracking performance of inside-out marker-based HMD-VR with optoelectronic systems. For example, Carnevale et al. [81] tracked translational (mean absolute error of 1.35 ± 0.66 cm) and rotational (mean absolute error of 1.11 ± 0.37°) shoulder movements. Also, Jost et al. [82] analysed positional data to determine the translational and rotational accuracy of the HMD (0.17 ± 0.07 cm, 0.34 ± 0.38°) and controllers (0.44 ± 0.29 cm, 1.13 ± 1.23°). Similarly, Monica and Aleotti [75] found an average translation error for the HMD of about 1.83 cm and an average rotation error of 0.77°.

Casile et al. [72] evaluated upper limb movements in post-stroke patients, comparing the inside-out markerless HMD-VR with optoelectronic marker-based MoCap. In particular, the authors found a slope close to 1 (maximum distance: 0.94 ± 0.1; peak velocity: 1.06 ± 0.12) from a linear regression analysis of peak velocity and hand positional data.

Furthermore, other authors compared the tracking performances of HMD-VR inside-out markerless platforms with IMUs. For instance, Trinidad-Fernandez et al. [20] calculated the absolute error of HMD (0.48 ± 0.09°), while Rojo et al. [71] compared controllers’ tracking accuracy to measure the elbow’s motion in the sagittal plane (intra-rater reliability of 0.999, with a 95% confidence interval).

Moreover, there is another issue to be considered for the implementation of HMD-VR in current clinical practice that is referred to regulatory considerations. In fact, CE marking indicates that a product has been assessed by the manufacturer and deemed to meet European safety, health, and environmental protection requirements. While this process is relatively short worldwide, in Europe, new laws have complicated this pathway. Obtaining a CE marking is a long and complex path that initially requires the recognition of the device as a medical one, and the Medical Device Regulation must be applicable. So the path to CE marking is long, time-consuming, and money-consuming, and this could hinder investments in innovation systems, like HMD-VR, for rehabilitation [83].

On the other hand, the European Union is allocating sustained funding for rehabilitation technologies, displacing private investments while contributing to the reconstruction of a more environmentally friendly, technologically advanced Europe. The World Health Organization (WHO) acknowledges the significance of health financing as a vital tool in advancing objectives such as enhancing accessibility, shielding against financial burdens, and facilitating the delivery of high-quality services. The WHO proposes that health financing strategies can be leveraged to advance these objectives specifically for rehabilitation services, thus allowing access to new technological devices [83].

## 5. Discussion

In this perspective review, we primarily aim to highlight the benefits and challenges of the different types of HMD-VR for the motion analysis of upper limbs in post-stroke patients, as compared to conventional MoCap systems. We identified the most used tracking technologies in clinical practice, encompassing their main technical features and comparing them to the novel HMD-VR. Despite the wide range of literature about the use of optoelectronics and IMUs for the motion analysis of upper limbs [8,29,48,49], few studies have, to date, dealt with HMD-VR technologies [50,66,72,81,82]. It seems that HMD-VR has been largely used as a tool for rehabilitation of upper limbs [19,80], but its potential use for motion analysis is still overlooked. Some authors used HMD-VR to assess visual neglect and motor performance in patients with Parkinson’s disease, demonstrating that HMD–VR allows instrumental quantitative and objective measurements [84].

In the context of upper-limb rehabilitation, reliable motion tracking is essential for effective rehabilitation [28]. In particular, hand tracking has a significant impact on the interaction between the patient and the virtual targets. Indeed, these systems should be able to recognise the individual differences among the different types of grips and range of movement of each individual patient [4,29,35]. Another point is that the VR rehabilitation systems must be able to record all data generated during the performance of the exercises. These data allow therapists to objectively assess the patient’s progress over time.

### 5.1. Benefits and Challenges of HMD-VR in a Neurorehabilitation Context

Differently from conventional tracking systems, HMD-VR platforms exploit the benefits of VR. They offer simultaneous task-oriented training and assessment within a controlled and safe environment that closely resembles real-world scenarios. Therefore, patients can perform ADL (cooking, driving, etc.) without any risk, in the simulated scenarios, through different levels of difficulty for each task [19]. A key benefit of using HMD-VR for motor assessment is the motivation of the patients. As already stated by Saldana et al. [19], these systems increase the patients’ motivation and engagement during rehabilitation tasks. Fostering patients’ intrinsic motivation to actively participate in the therapeutic process is especially important, particularly when conventional methods may lead to boredom or lack of interest [85]. The available studies [22,85,86] on neurorehabilitation indicate that HMD-VR exhibits the potential to enhance tailored assessment and intervention methods by engaging patients within an immersive virtual environment. According to different authors [22,36,86,87], HMD-VR can be an effective tool to improve upper limb motor outcomes and kinematic factors, as well as ADLs, in post-stroke patients. In particular, De Giorgi et al. [88] used HMD-VR to perform virtual art therapy, which remarkably promotes the autonomy of post-stroke patients in their ADLs and upper limb functioning, in terms of muscle strength and pinching.

Furthermore, HMD-VR platforms were perceived as easy to use, and user-friendly by post-stroke patients. Some authors reported high levels of usability, and adherence to the treatment [87,89], which could have positively influenced their intention in using the VR exercise system. In this way, the authors enable multisensorial stimulation, involving auditory, visual, and tactile feedback, to promote patients’ engagement.

To enhance active participation and adherence to the treatment, Rodrigues et al. [65] proposed a prototype of combined augmented reality interface with collaborative arm robot. This integrated method, combining augmented reality with a robotic arm, enables the creation of novel user experiences by harnessing gamification principles. These include introducing challenging scenarios, implementing reward systems, and even encouraging users to surpass their previous high scores. Collaborative robots are growing in popularity as translational devices, and they can be used as a valid and alternative to VR tools, especially in those patients suffering from cybersickness. In rehabilitation, they can be used in active or passive mode. In active mode, they can guide the patients in the early stages when they need to learn (relearning mechanism). Gradually, they can be used in passive mode where the patients are guiding the robot arm. In addition, they also provide kinematic information about a patient’s performance [90].

Like any other therapy, rehabilitation with HMD-VR has its limitations and challenges. In fact, it should be also considered that these systems can elicit cybersickness (including dizziness, headaches, disorientation, and fatigue) as well as side-effects which may limit the usability of HMD-VR. Some studies have shown that side effects tend to get worse in full VR immersion, having a negative effect on static balance [91]. According to Caserman et al. [66], some participants reported cybersickness, such as dizziness, nausea, or a headache, while wearing an HMD for a longer time. Another side-effect that could be elicited by immersive VR is the loss of visual contact within the environment and rehabilitation setting (including physiotherapists), with potential negative consequences for patients’ psychological state.

Despite the limited evidence on the side effects of HMD-VR, this must be considered when choosing the type and the duration of rehabilitation for a neurological patient. In addition, it should be considered that post-stroke patients may suffer from psychological conditions, like anxiety and depression, which could overload the sickness effects provided by the VR. This is the case when it is recommended to use other devices, including robots and cobots. However, a previous case study [92] reported that rehabilitation in an immersive VR environment, in a post-stroke subject, was effective in improving psychological symptoms. In this sense, the authors spelt out the use of immersive VR as a promising device for the of psychological post-stroke symptoms, including anxiety.

Another clinical challenge for the implementation of HMD-VR in post-stroke rehabilitation consists of some difficulties when the subject is managing the technology [24]. Indeed, interaction with HMD-VR requires a long press of the button on the controller, which can be demanding for post-stroke subjects. To this aim, patients could benefit from robotic-assisted upper limbs training, which supports the paretic limb against throughout the RoM required by the exercise, preventing the subject from becoming discouraged due to muscle weakness. In contrast to VR, robotic devices can allow the mobilisation of the hand and fingers, also giving a physiological trajectory of movement while avoiding abnormal muscle compensations due to spasticity.

However, robotic devices can require a long time for setup and specialised staff to operate with them. In fact, precise alignment between users and robotic devices is crucial to prevent adverse interaction forces that could lead to discomfort and safety concerns. To overcome this issue, soft exoskeletons, composed of flexible textiles or elastomers, present a promising solution by enhancing user compliance compared to rigid orthoses. However, the limited availability of robotic devices in rehabilitation centres due to cost, maintenance, and specialised staff requirements poses a significant obstacle to their integration into clinical practice at scale. These factors contribute to the current limited adoption of robot-based assessments in clinical settings.

On the other hand, HMD-VR exploits the conventional tracking methodology of these technologies by cutting down on costs and complexity, thanks to their link with the world of consumer electronics. The accessibility of these devices should help make future studies using HMD-VR with larger populations more feasible. Considering that HMD-VR represents a growing and potential field, it should be also analysed in terms of cost-effectiveness. The whole HMD-VR platform (HMD, computer, and/or controllers, and/or lighthouses) is relatively low-cost when compared with other VR devices [93]. In addition, the primary savings due to the use of HMD-VR for post-stroke patients rely on its utilisation for both evaluation and treatment with or without the presence of the therapist. In this latter condition, HMD-VR allows for the reduction in costs related to therapist’s service and travel expenses for both the therapist (i.e., domiciliary therapy) and the caregiver (i.e., ambulatory care) in terms of money, time spent, and effort. To this aim, some authors [94] investigated the effects of an outside-in HMD-VR for telerehabilitation, in a post-stroke patient. Although this is only one case study, the authors have shown that HMD-VR can be an ecological, user-friendly, and adjunctive rehabilitation approach to conventional treatment, even for those subjects living in rural regions.

### 5.2. Reliability, Technical Features, and Limitations of HMD-VR

On the other hand, the analysis of motion in the neurorehabilitation context allows the instrumental registration and monitoring of patients’ movement parameters (Table 1) in a more objective way than clinical scales (clinical purpose). The multitude of data produced from motion analysis can be used to build machine learning algorithms, detecting prognostic and predictive factors for better motor outcomes (research purpose) [5].

To this end, both accuracy and precision of tracking measurements are essential for obtaining reliable results. Indeed, many studies have focused on the tracking measurement quality of such systems compared with conventional MoCap systems. For instance, Nie et al. [50] and Caserman et al. [66] found that outside-in HMD-VR devices (HTC Vive) track the wrist during reaching, with mean signed errors of 0.48 ± 1.58 cm (36) and a latency of 6.71 ± 0.80 ms (42) when compared to a traditional optical tracking system. According to Nie et al. [50], the IMUs and HMD-VR (HTC Vive) necessitate minimal alignment and calibration steps compared to optical tracking systems like Vicon. These procedures take at most 10 min and can be performed by individuals, including those with stroke. In particular, Caserman et al. [66] reported encouraging results on the accuracy of the HMD (HTC Vive). Indeed, the system efficiently provided real-time joint configuration, enabling smooth and accurate movement tracking.

Regarding inside-out marker-based device (Oculus Quest 2), Carnevale et al. [81] measured the rotational and translational movements of the upper limbs with a mean absolute error of 1.11 ± 0.37° and 13.52 ± 6.57 mm, respectively (51). These results were registered at 500 mm from the HMD along the x-direction. On the other hand, a recent study [82] investigating the accuracy of the same inside-out marker-based device reported a reduced absolute error. The researchers [82] pointed out that the best controller performance occurred when the HMD wearer observed it closely, which contrasts with Carnevale’s et al. [81] study where they varied the distance between the headset and controller.

In contrast to previous studies, Rojo et al. [71] found a high agreement level of 0.999 (with a 95% confidence interval) between measurements of forearm flexion-extension using both Oculus Touch v2 controllers and IMU devices. However, the controllers exhibit inaccuracies, especially near a 90° angle in the sagittal rotation plane. Despite this, they adequately capture the full range of motion of the elbow joint in virtual environments. According to the authors [71], while minor misalignments may be acceptable in VR applications where precision is not critical, for tasks requiring high accuracy in orientation measurement, an IMU sensor is preferable.

Finally, comparing markerless inside-out HMD-VR with optoelectronic systems, Casile et al. [72] found a maximum distance of 0.94 ± 0.1 and a peak velocity of 1.06 ± 0.12. The authors suggested that the inside-out markerless HMD-VR (Oculus Quest) provided hand position and peak velocity estimates that closely matched those of a marker-based commercial system. In addition, Casile et al. found that Oculus Quest was also sensitive in distinguishing pathological from healthy upper limb movements.

From a technical point of view, HMDs present some differences and limitations among them. For example, the outside-in systems enable the estimation of upper limb during complex tasks, even when they are outside of the field of view of the HMD. In contrast, inside-out can only estimate the position of the upper limbs if they are within the field of view of the HMD cameras [75]. Additionally, outside-in systems are generally faster and more accurate than inside-out systems, and the accuracy of these systems can be easily improved by adding more cameras. Also, outside-in systems are capable of tracking even in dark settings, and controllers can be tracked even while they are behind the user’s back [75]. Indeed, inside-out systems cannot estimate the position of the patient’s paretic hand if it is outside the field of view of HMD cameras during a reaching task of an object, framed with the HMD. These systems are easier to set up than outside-in, since there is no need to install fixed cameras for the calibration. Regardless of whether these systems are inside-out or outside-in, marker-based systems are generally less reliable in estimating hand gestures [14]. In fact, these systems employ a controller, enabling the estimation of a few basic hand gestures decoded through the controller’s buttons. Conversely, the HMD-VR inside-out markerless systems facilitate complex hand gesture recognition but necessitate computer vision algorithms. It is noteworthy that the HMD-VR platform is based on conventional and validated MoCap methods to estimate user’s motion. Conventional methods are accurate although they present some disadvantages related to their expensiveness, which are time-consuming for instrumentation and data processing, requiring qualified staff to be used in some cases [10]. In addition, these systems have not yet been recognised as medical devices; in fact, they do not have the CE mark. However, their use in the world of gaming and education has been translated to the neurorehabilitation field. Overall, these aspects could explain why these devices are not always used in clinical settings, but they are largely diffused in the context of research.

### 5.3. Clinical Perspectives and Future Directions

From a clinical point of view, the motion analysis of hand gesture is fundamental to track those compensatory strategies that post-stroke patients use to perform a grasping function. Some examples refer to reduced finger abduction, proximal interphalangeal joint flexion, and metacarpophalangeal joint extension during object grasping [28,29,48]. To overcome these issues, rehabilitation modalities should ideally be affordable, easily transportable, and user-friendly for long-term use. Specifically, portability and ease of use are crucial for promoting treatment adherence and enabling continuous care at home. While full embodiment in a virtual avatar may not be necessary, the presence of the affected body part in the virtual environment is essential. Creating a feeling of presence by synchronising limb movements in real-time is crucial, and monitoring both the affected and intact limbs may be necessary for the task. Therefore, HMD-VR platforms meet most of the needs of post-stroke patients, both in terms of treatment and kinematic motion analysis. However, the dose of treatment and which patients to select is still to be understood. Researchers and clinicians must consider the risks faced by certain individuals when engaging in VR. Examples include those with epilepsy, seizure disorders, recent concussions, and elderly adults experiencing vision impairment due to age-related changes. These groups may encounter adverse effects during VR experiences, highlighting the importance of clinical patients’ examination.

In the end, we could hypothesise that HMD-VR should be considered task-specific-based. This aspect has an important bearing on clinical practice, because the choice of HMD-VR is closely linked to the type of task that needs to be assessed. Clinicians need to consider their intended use and what they want to achieve, in terms of reliability and effectiveness. To this aim, the collaboration between clinicians bioengineering professional figures should be also considered. This aspect can promote the implementation of innovative technologies, like HMD-VR, in the neurorehabilitation setting. For example, physiotherapists and neurologists could collaborate more with bioengineers to share bidirectionally practical information, which is needed to plan specific motor training based on objective and quantitative findings.

Indeed, since these devices are being designed for the gaming/entertainment world, the newest models may not always be the best solution in the rehabilitation field, especially in neurological patients. Future research and clinical trials aiming to validate the effectiveness of HMD-VR technology in rehabilitation may involve investigating the accuracy of both outside-in and inside-out HMD-VR systems compared to conventional MoCap methods across various motor tasks. Another future consideration is the utilisation of these systems at home for both treatment and kinematic motion analysis, ensuring the continuity of care. Additionally, the extensive data collected from these devices could be leveraged to develop machine learning algorithms capable of predicting outcomes. Finally, we present some perspectives about the use of HMD-VR platform for upper limb motion estimation in neurorehabilitation context. From our literature analysis, we gather the following points:

- A comparison between the different types and brands of HMD-VR platforms is still missing, intended as measurement and rehabilitation tools. In addition, among the studies that we have included in this review, there is no homogeneity in results regarding accuracy and precision analysis.
- According to the authors (Table 4), these systems are suitably accurate and reliable to be used as rehabilitation tools and MoCap systems.
- The selection of one device over another depends on its intended use and on the severity degree of the disease. Therefore, clinicians must consider the reliability and effectiveness of the instrument.
- It is noteworthy that VR is not the only technology capable of providing an objective and quantitative assessment of movement as well as delivering rehabilitation treatment. In fact, robotic devices can be tailored to meet the patient’s needs, offering both precise movement evaluation and intensive, repetitive, and task-oriented treatment options.

## 6. Conclusions

According to the reviewed literature, the HMD-VR platform can be a safe, cost-effective, and portable tool for upper limb kinematic motion analysis in the context of neurorehabilitation. The HMD-VR platforms not only provide the advantage of offering an objective and quantitative assessment of upper limb function but also support rehabilitation treatment. Particularly for post-stroke patients, VR presents a compelling option due to its immersive and safe environment enriched with multisensory stimuli. This immersive experience enhances patient attention, fostering increased engagement and adherence to treatment protocols. Yet, it is crucial to consider both the duration of treatment and the severity of patients’ conditions when formulating rehabilitation plans, taking into consideration also other devices, including robots and cobots. Indeed, the use of VR is still far from being considered standard in clinical practice. Future studies should explore HMD–VR applications in neurological clinical settings and home-based training to better investigate whether and to which extent these promising tools can be used in the movement assessment of patients with neurological disorders, including stroke.

## Figures and Tables

**Table 1:** The main kinematic features extracted from motion analysis of the upper limb in post-stroke patients.

Kinematic Features	Description
Path length ratio	Path length ratio (PLR) in upper limb kinematic analysis is a quantitative measure used to assess movement efficiency. It is calculated by dividing the actual path length travelled by a specific point or segment of the upper limb during a movement task by the shortest possible path length for the same movement. Higher PLR indicates less efficient movement patterns.
Joint excursion	Joint excursion consists of the angular RoM experienced by a specific joint during a movement task. It is typically measured in degrees or radians and provides insight into the flexibility, mobility, and coordination of the joint during the execution of a motor task.
Smoothness	Smoothness in upper limb kinematic analysis is a quantitative measure used to assess movement quality during reaching. There are several methods to measure movement smoothness. One of these is calculated as the number of peaks detected in the velocity profile.
Movement time	Movement time refers to the duration taken to complete a specific movement task. It is a crucial measure in assessing motor control, coordination, and efficiency of upper limb movements.
Movement velocity	Movement velocity is calculated by dividing the displacement of the point or segment by the time taken to complete the movement. It provides information about the speed or pace of movement execution, and it can be used to understand motor performance, coordination dynamics, and task difficulty.
Peak velocity	Peak velocity refers to the maximum instantaneous velocity achieved by a specific point or segment of the upper limb during a movement task. It represents the highest speed attained during the movement.
Number of velocity peaks	The number of velocity peaks refers to the count of distinct instances where the velocity of a specific point or segment of the upper limb reaches a local maximum during a movement task. Each velocity peak corresponds to a moment of rapid acceleration or deceleration within the movement profile.

**Table 2:** Shows a schematic description of the advantages and limitations of HMD-VR systems relative to conventional MoCap and robot-based systems used for upper limb kinematic motion analysis.

MoCap System	Portability	Markerless	Easy-to-Use	Tele-Monitoring	Untethered	Rehabilitation	Cost
Optoelectronic	No	No	No	No	No	No	High
IMU	Yes	No	Yes	Yes	No	No	Medium
Vision-based	Yes	Yes	Yes	Yes	No	No	Low
Robot	No	Yes	No	No	No	Yes	High
HMD-VR	Yes	Yes	Yes	Yes	Yes	Yes	Low

**Table 3:** Table shows the differences/meeting points between outside-in and inside-out, both marker-based and markerless.

Features	Outside-In	Inside-Out (Marker-Based)	Inside-Out (Markerless)
Portability	✗	✓	✓
Untethered	✗	✓	✓
Hand gesture	✗	✗	✓
HMD field of view tracking	✓	✓	✓
External field of view tracking	✓	✗	✗

**Table 4:** Description of the most significant studies evaluating accuracy of HMD-VR for upper limb position estimation.

Study Reference	MoCap Systems Comparison	Performed Task	Parameters of Precision/Accuracy	Kinematic Assessment
HMD Outside-In				
[50]	IMUs and HMD VR sensor (Vive) compared with Optoelectronic system (Vicon)	Wrist position, during reaching tasks, with respect to the shoulder	Compared to a traditional optical tracking system, both methods accurately tracked the wrist during reaching, with mean signed errors of 0.09 ± 1.81 cm and 0.48 ± 1.58 cm for the IMUs and Vive, respectively.	Normalised mean endpoint speed (Smoothness)
[66]	HTC Vive HMD and Vive tracker	Reaching tasks	HTC Vive headset and Vive Trackers showed that both can track joint rotation and position with reasonable accuracy and a very low end to latency of 6.71 ± 0.80 ms.	Joint rotation and position
HMD Inside-Out Marker-based				
[81]	HMD (Oculus Quest 2) compared with Qualysis optical capture system	Upper limb rotational and translational movements	The results showed a mean absolute error of 13.52 ± 6.57 mm at a distance of 500 mm from the HMD along the x-direction. The maximum mean absolute error for rotational displacements was found to be 1.11 ± 0.37° for a rotation of 40° around the *z*-axis.	Translational and rotational movement
[71]	HMD (Oculus Touch v2) controller compared with IMU	Flexion–extension movement of the forearm.	The level of agreement between the measurements of these devices was 0.999 with a 95% confidence interval (ranged from 0.996 to 1.000). The accuracy degrades at flexion values between 70° and 110°, peaking at 90°.	Range of motion of elbow in the sagittal plane
HMD Inside-Out Marker-less				
[72]	HMD (Oculus Quest 2, Meta) compared with Optoelectronic system (Optitrack).	Reaching	Maximum distance: mean slope = 0.94 ± 0.1; peak velocity: mean slope = 1.06 ± 0.12).	Peak velocity and hand position

## Data Availability

Not applicable.

## References

[1] Anwer S., Waris A., Gilani S.O., Iqbal J., Shaikh N., Pujari A.N., Niazi I.K. (2022). Rehabilitation of Upper Limb Motor Impairment in Stroke: A Narrative Review on the Prevalence, Risk Factors, and Economic Statistics of Stroke and State of the Art Therapies. Healthcare.

[2] Wafa H.A., Wolfe C.D.A., Emmett E., Roth G.A., Johnson C.O., Wang Y. (2020). Burden of Stroke in Europe: Thirty-Year Projections of Incidence, Prevalence, Deaths, and Disability-Adjusted Life Years. Stroke.

[3] Murphy S.J.X., Werring D.J. (2020). Stroke: Causes and Clinical Features. Medicine.

[4] Schwarz A., Bhagubai M.M.C., Nies S.H.G., Held J.P.O., Veltink P.H., Buurke J.H., Luft A.R. (2022). Characterization of Stroke-Related Upper Limb Motor Impairments across Various Upper Limb Activities by Use of Kinematic Core Set Measures. J. NeuroEngineering Rehabil..

[5] Bonanno M., Calabrò R.S. (2023). Robot-Aided Motion Analysis in Neurorehabilitation: Benefits and Challenges. Diagnostics.

[6] Chiriatti G., Bottiglione A., Palmieri G. (2022). Manipulability Optimization of a Rehabilitative Collaborative Robotic System. Machines.

[7] Pan B., Huang Z., Jin T., Wu J., Zhang Z., Shen Y. (2021). Motor Function Assessment of Upper Limb in Stroke Patients. J. Healthc. Eng..

[8] Gu C., Lin W., He X., Zhang L., Zhang M. (2023). IMU-Based Motion Capture System for Rehabilitation Applications: A Systematic Review. Biomim. Intell. Robot..

[9] Topley M., Richards J.G. (2020). A Comparison of Currently Available Optoelectronic Motion Capture Systems. J. Biomech..

[10] van Schaik J.E., Dominici N., Hunnius S., Meyer M. (2020). Chapter 5—Motion Tracking in Developmental Research: Methods, Considerations, and Applications. Progress in Brain Research.

[11] Zago M., Luzzago M., Marangoni T., De Cecco M., Tarabini M., Galli M. (2020). 3D Tracking of Human Motion Using Visual Skeletonization and Stereoscopic Vision. Front. Bioeng. Biotechnol..

[12] Aguilera-Rubio Á., Alguacil-Diego I.M., Mallo-López A., Cuesta-Gómez A. (2022). Use of the Leap Motion Controller^®^ System in the Rehabilitation of the Upper Limb in Stroke. A Systematic Review. J. Stroke Cerebrovasc. Dis..

[13] Potter M.V., Cain S.M., Ojeda L.V., Gurchiek R.D., McGinnis R.S., Perkins N.C. (2022). Evaluation of Error-State Kalman Filter Method for Estimating Human Lower-Limb Kinematics during Various Walking Gaits. Sensors.

[14] Colyer S.L., Evans M., Cosker D.P., Salo A.I.T. (2018). A Review of the Evolution of Vision-Based Motion Analysis and the Integration of Advanced Computer Vision Methods Towards Developing a Markerless System. Sports Med. Open.

[15] Gibbs J.K., Gillies M., Pan X. (2022). A Comparison of the Effects of Haptic and Visual Feedback on Presence in Virtual Reality. Int. J. Hum. Comput. Stud..

[16] Lindeman R.W., Page R., Yanagida Y., Sibert J.L. Towards Full-Body Haptic Feedback: The Design and Deployment of a Spatialized Vibrotactile Feedback System. Proceedings of the ACM Symposium on Virtual Reality Software and Technology.

[17] Pacchierotti C., Sinclair S., Solazzi M., Frisoli A., Hayward V., Prattichizzo D. (2017). Wearable Haptic Systems for the Fingertip and the Hand: Taxonomy, Review, and Perspectives. IEEE Trans. Haptics.

[18] Li F., Chen J., Ye G., Dong S., Gao Z., Zhou Y. (2023). Soft Robotic Glove with Sensing and Force Feedback for Rehabilitation in Virtual Reality. Biomimetics.

[19] Saldana D., Neureither M., Schmiesing A., Jahng E., Kysh L., Roll S.C., Liew S.-L. (2020). Applications of Head-Mounted Displays for Virtual Reality in Adult Physical Rehabilitation: A Scoping Review. Am. J. Occup. Ther..

[20] Trinidad-Fernández M., Bossavit B., Salgado-Fernández J., Abbate-Chica S., Fernández-Leiva A.J., Cuesta-Vargas A.I. (2023). Head-Mounted Display for Clinical Evaluation of Neck Movement Validation with Meta Quest 2. Sensors.

[21] Fregna G., Paoluzzi C., Baroni A., Cano-de-la-Cuerda R., Casile A., Straudi S. (2023). Head-Mounted Displays for Upper Limb Stroke Rehabilitation: A Scoping Review. J. Clin. Med..

[22] Kiper P., Godart N., Cavalier M., Berard C., Cieślik B., Federico S., Kiper A., Pellicciari L., Meroni R. (2024). Effects of Immersive Virtual Reality on Upper-Extremity Stroke Rehabilitation: A Systematic Review with Meta-Analysis. J. Clin. Med..

[23] Amini Gougeh R., Falk T.H. (2022). Head-Mounted Display-Based Virtual Reality and Physiological Computing for Stroke Rehabilitation: A Systematic Review. Front. Virtual Real..

[24] Marek K., Zubrycki I., Miller E. (2022). Immersion Therapy with Head-Mounted Display for Rehabilitation of the Upper Limb after Stroke—Review. Sensors.

[25] Kwakkel G., Van Wegen E., Burridge J.H., Winstein C.J., van Dokkum L., Alt Murphy M., Levin M.F., Krakauer J.W. (2019). Standardized Measurement of Quality of Upper Limb Movement after Stroke: Consensus-Based Core Recommendations from the Second Stroke Recovery and Rehabilitation Roundtable. Int. J. Stroke.

[26] Duprey S., Naaim A., Moissenet F., Begon M., Chèze L. (2017). Kinematic Models of the Upper Limb Joints for Multibody Kinematics Optimisation: An Overview. J. Biomech..

[27] De Baets L., Vanbrabant S., Dierickx C., van der Straaten R., Timmermans A. (2020). Assessment of Scapulothoracic, Glenohumeral, and Elbow Motion in Adhesive Capsulitis by Means of Inertial Sensor Technology: A Within-Session, Intra-Operator and Inter-Operator Reliability and Agreement Study. Sensors.

[28] Schwarz A., Bhagubai M.M.C., Wolterink G., Held J.P.O., Luft A.R., Veltink P.H. (2020). Assessment of Upper Limb Movement Impairments after Stroke Using Wearable Inertial Sensing. Sensors.

[29] Schwarz A., Kanzler C.M., Lambercy O., Luft A.R., Veerbeek J.M. (2019). Systematic Review on Kinematic Assessments of Upper Limb Movements After Stroke. Stroke.

[30] Valtin M., Salchow C., Seel T., Laidig D., Schauer T. (2017). Modular Finger and Hand Motion Capturing System Based on Inertial and Magnetic Sensors. Curr. Dir. Biomed. Eng..

[31] Mesquita I.A., da Fonseca P.F.P., Pinheiro A.R.V., Velhote Correia M.F.P., da Silva C.I.C. (2019). Methodological Considerations for Kinematic Analysis of Upper Limbs in Healthy and Poststroke Adults Part II: A Systematic Review of Motion Capture Systems and Kinematic Metrics. Top. Stroke Rehabil..

[32] Bernhardt J., Hayward K.S., Dancause N., Lannin N.A., Ward N.S., Nudo R.J., Farrin A., Churilov L., Boyd L.A., Jones T.A. (2019). A Stroke Recovery Trial Development Framework: Consensus-Based Core Recommendations from the Second Stroke Recovery and Rehabilitation Roundtable. Int. J. Stroke.

[33] Prange-Lasonder G.B., Alt Murphy M., Lamers I., Hughes A.-M., Buurke J.H., Feys P., Keller T., Klamroth-Marganska V., Tarkka I.M., Timmermans A. (2021). European Evidence-Based Recommendations for Clinical Assessment of Upper Limb in Neurorehabilitation (CAULIN): Data Synthesis from Systematic Reviews, Clinical Practice Guidelines and Expert Consensus. J. NeuroEngineering Rehabil..

[34] Johansson G.M., Grip H., Levin M.F., Häger C.K. (2017). The Added Value of Kinematic Evaluation of the Timed Finger-to-Nose Test in Persons Post-Stroke. J. NeuroEngineering Rehabil..

[35] van der Vliet R., Selles R.W., Andrinopoulou E.-R., Nijland R., Ribbers G.M., Frens M.A., Meskers C., Kwakkel G. (2020). Predicting Upper Limb Motor Impairment Recovery after Stroke: A Mixture Model. Ann. Neurol..

[36] Huang X., Liao O., Jiang S., Li J., Ma X. (2024). Kinematic Analysis in Post-Stroke Patients with Moderate to Severe Upper Limb Paresis and Non-Disabled Controls. Clin. Biomech..

[37] Choi H., Park D., Rha D.-W., Nam H.S., Jo Y.J., Kim D.Y. (2023). Kinematic Analysis of Movement Patterns during a Reach-and-Grasp Task in Stroke Patients. Front. Neurol..

[38] Guzik-Kopyto A., Nowakowska-Lipiec K., Krysiak M., Jochymczyk-Woźniak K., Jurkojć J., Wodarski P., Gzik M., Michnik R. (2022). Selection of Kinematic and Temporal Input Parameters to Define a Novel Upper Body Index Indicator for the Evaluation of Upper Limb Pathology. Appl. Sci..

[39] Aprile I., Rabuffetti M., Padua L., Di Sipio E., Simbolotti C., Ferrarin M. (2014). Kinematic Analysis of the Upper Limb Motor Strategies in Stroke Patients as a Tool towards Advanced Neurorehabilitation Strategies: A Preliminary Study. Biomed. Res. Int..

[40] Flex 13-In Depth. http://optitrack.com/cameras/flex-13/index.html.

[41] MVN Awinda|Movella.Com. https://www.movella.com/products/motion-capture/xsens-mvn-awinda.

[42] Wade L., Needham L., McGuigan P., Bilzon J. (2022). Applications and Limitations of Current Markerless Motion Capture Methods for Clinical Gait Biomechanics. PeerJ.

[43] Boser Q.A., Valevicius A.M., Lavoie E.B., Chapman C.S., Pilarski P.M., Hebert J.S., Vette A.H. (2018). Cluster-Based Upper Body Marker Models for Three-Dimensional Kinematic Analysis: Comparison with an Anatomical Model and Reliability Analysis. J. Biomech..

[44] Bodily K.D., Daniel T.A., Sturz B.R. (2012). The Roles of Beaconing and Dead Reckoning in Human Virtual Navigation. Learn. Motiv..

[45] Ponsiglione A.M., Ricciardi C., Amato F., Cesarelli M., Cesarelli G., D’Addio G. (2022). Statistical Analysis and Kinematic Assessment of Upper Limb Reaching Task in Parkinson’s Disease. Sensors.

[46] Held J.P.O., Klaassen B., Eenhoorn A., van Beijnum B.-J.F., Buurke J.H., Veltink P.H., Luft A.R. (2018). Inertial Sensor Measurements of Upper-Limb Kinematics in Stroke Patients in Clinic and Home Environment. Front. Bioeng. Biotechnol..

[47] Zhou L., Fischer E., Tunca C., Brahms C.M., Ersoy C., Granacher U., Arnrich B. (2020). How We Found Our IMU: Guidelines to IMU Selection and a Comparison of Seven IMUs for Pervasive Healthcare Applications. Sensors.

[48] Merlau B., Cormier C., Alaux A., Morin M., Montané E., Amarantini D., Gasq D. (2023). Assessing Spatiotemporal and Quality Alterations in Paretic Upper Limb Movements after Stroke in Routine Care: Proposal and Validation of a Protocol Using IMUs versus MoCap. Sensors.

[49] Unger T., de Sousa Ribeiro R., Mokni M., Weikert T., Pohl J., Schwarz A., Held J.P.O., Sauerzopf L., Kühnis B., Gavagnin E. (2024). Upper Limb Movement Quality Measures: Comparing IMUs and Optical Motion Capture in Stroke Patients Performing a Drinking Task. Front. Digit. Health.

[50] Nie J.Z., Nie J.W., Hung N.-T., Cotton R.J., Slutzky M.W. (2021). Portable, Open-Source Solutions for Estimating Wrist Position during Reaching in People with Stroke. Sci. Rep..

[51] GitHub-Google-Ai-Edge/Mediapipe: Cross-Platform, Customizable ML Solutions for Live and Streaming Media. https://github.com/google-ai-edge/mediapipe.

[52] MoveNet: Modello di Rilevamento Della Posa Ultra Veloce e Preciso. TensorFlow Hub. https://www.tensorflow.org/hub/tutorials/movenet?hl=it.

[53] CMU-Perceptual-Computing-Lab/Openpose: OpenPose: Real-Time Multi-Person Keypoint Detection Library for Body, Face, Hands, and Foot Estimation. https://github.com/CMU-Perceptual-Computing-Lab/openpose.

[54] Ceseracciu E., Sawacha Z., Cobelli C. (2014). Comparison of Markerless and Marker-Based Motion Capture Technologies through Simultaneous Data Collection during Gait: Proof of Concept. PLoS ONE.

[55] Faity G., Mottet D., Froger J. (2022). Validity and Reliability of Kinect v2 for Quantifying Upper Body Kinematics during Seated Reaching. Sensors.

[56] Galeoto G., Berardi A., Mangone M., Tufo L., Silvani M., González-Bernal J., Seco-Calvo J. (2023). Assessment Capacity of the Armeo^®^ Power: Cross-Sectional Study. Technologies.

[57] Merlo A., Longhi M., Giannotti E., Prati P., Giacobbi M., Ruscelli E., Mancini A., Ottaviani M., Montanari L., Mazzoli D. (2013). Upper Limb Evaluation with Robotic Exoskeleton. Normative Values for Indices of Accuracy, Speed and Smoothness. NeuroRehabilitation.

[58] Longhi M., Merlo A., Prati P., Giacobbi M., Mazzoli D. (2016). Instrumental Indices for Upper Limb Function Assessment in Stroke Patients: A Validation Study. J. NeuroEng. Rehabil..

[59] Schweighofer N., Choi Y., Winstein C., Gordon J. (2012). Task-Oriented Rehabilitation Robotics. Am. J. Phys. Med. Rehabil..

[60] Maciejasz P., Eschweiler J., Gerlach-Hahn K., Jansen-Troy A., Leonhardt S. (2014). A Survey on Robotic Devices for Upper Limb Rehabilitation. J. NeuroEngineering Rehabil..

[61] Wagner T.H., Lo A.C., Peduzzi P., Bravata D.M., Huang G.D., Krebs H.I., Ringer R.J., Federman D.G., Richards L.G., Haselkorn J.K. (2011). An Economic Analysis of Robot-Assisted Therapy for Long-Term Upper-Limb Impairment after Stroke. Stroke.

[62] Cherubini A., Navarro-Alarcon D. (2021). Sensor-Based Control for Collaborative Robots: Fundamentals, Challenges, and Opportunities. Front. Neurorobot..

[63] Kyrkjebø E., Johan Laastad M., Stavdahl Ø. Feasibility of the UR5 Industrial Robot for Robotic Rehabilitation of the Upper Limbs After Stroke. Proceedings of the 2018 IEEE/RSJ International Conference on Intelligent Robots and Systems (IROS).

[64] Reinkensmeyer D.J., Wolbrecht E.T., Chan V., Chou C., Cramer S.C., Bobrow J.E. (2012). Comparison of 3D, Assist-as-Needed Robotic Arm/Hand Movement Training Provided with Pneu-WREX to Conventional Table Top Therapy Following Chronic Stroke. Am. J. Phys. Med. Rehabil..

[65] Rodrigues J.C., Menezes P., Restivo M.T. (2023). An Augmented Reality Interface to Control a Collaborative Robot in Rehab: A Preliminary Usability Evaluation. Front. Digit. Health.

[66] Caserman P., Garcia-Agundez A., Konrad R., Göbel S., Steinmetz R. (2019). Real-Time Body Tracking in Virtual Reality Using a Vive Tracker. Virtual Real..

[67] Pandita S., Stevenson Won A., Kim J., Song H. (2020). Chapter 7—Clinical Applications of Virtual Reality in Patient-Centered Care. Technology and Health.

[68] Campo-Prieto P., Cancela-Carral J.M., Rodríguez-Fuentes G. (2022). Wearable Immersive Virtual Reality Device for Promoting Physical Activity in Parkinson’s Disease Patients. Sensors.

[69] Varela-Aldás J., Palacios-Navarro G., Amariglio R., García-Magariño I. (2020). Head-Mounted Display-Based Application for Cognitive Training. Sensors.

[70] Memmesheimer V.M., Ebert A. (2022). Scalable Extended Reality: A Future Research Agenda. Big Data Cogn. Comput..

[71] Rojo A., Cortina J., Sánchez C., Urendes E., García-Carmona R., Raya R. (2022). Accuracy Study of the Oculus Touch v2 versus Inertial Sensor for a Single-Axis Rotation Simulating the Elbow’s Range of Motion. Virtual Real..

[72] Casile A., Fregna G., Boarini V., Paoluzzi C., Manfredini F., Lamberti N., Baroni A., Straudi S. (2023). Quantitative Comparison of Hand Kinematics Measured with a Markerless Commercial Head-Mounted Display and a Marker-Based Motion Capture System in Stroke Survivors. Sensors.

[73] Unity Real-Time Development Platform|3D, 2D, VR & AR Engine. https://unity.com/.

[74] Downloads-Meta XR Core SDK (UPM). https://developer.oculus.com/downloads/package/meta-xr-core-sdk/.

[75] Monica R., Aleotti J. (2022). Evaluation of the Oculus Rift S Tracking System in Room Scale Virtual Reality. Virtual Real..

[76] Pereira D., Oliveira V., Vilaça J.L., Carvalho V., Duque D. (2023). Measuring the Precision of the Oculus Quest 2′s Handheld Controllers. Actuators.

[77] Kharoub H., Lataifeh M., Ahmed N. (2019). 3D User Interface Design and Usability for Immersive VR. Appl. Sci..

[78] SteamVR su Steam. https://store.steampowered.com/app/250820/SteamVR/.

[79] Mirzaei B., Nezamabadi-pour H., Raoof A., Derakhshani R. (2023). Small Object Detection and Tracking: A Comprehensive Review. Sensors.

[80] Fregna G., Schincaglia N., Baroni A., Straudi S., Casile A. (2022). A Novel Immersive Virtual Reality Environment for the Motor Rehabilitation of Stroke Patients: A Feasibility Study. Front. Robot. AI.

[81] Carnevale A., Mannocchi I., Sassi M.S.H., Carli M., De Luca G., Longo U.G., Denaro V., Schena E. (2022). Virtual Reality for Shoulder Rehabilitation: Accuracy Evaluation of Oculus Quest 2. Sensors.

[82] Jost T.A., Nelson B., Rylander J. (2021). Quantitative Analysis of the Oculus Rift S in Controlled Movement. Disabil. Rehabil. Assist. Technol..

[83] Calabrò R.S., Müller-Eising C., Diliberti M.L., Manuli A., Parrinello F., Rao G., Barone V., Civello T. (2020). Who Will Pay for Robotic Rehabilitation? The Growing Need for a Cost-Effectiveness Analysis. Innov. Clin. Neurosci..

[84] Arias P., Robles-García V., Sanmartín G., Flores J., Cudeiro J. (2012). Virtual Reality as a Tool for Evaluation of Repetitive Rhythmic Movements in the Elderly and Parkinson’s Disease Patients. PLoS ONE.

[85] Aderinto N., Olatunji G., Abdulbasit M.O., Edun M., Aboderin G., Egbunu E. (2023). Exploring the Efficacy of Virtual Reality-Based Rehabilitation in Stroke: A Narrative Review of Current Evidence. Ann. Med..

[86] Demeco A., Zola L., Frizziero A., Martini C., Palumbo A., Foresti R., Buccino G., Costantino C. (2023). Immersive Virtual Reality in Post-Stroke Rehabilitation: A Systematic Review. Sensors.

[87] Chen X., Liu F., Lin S., Yu L., Lin R. (2022). Effects of Virtual Reality Rehabilitation Training on Cognitive Function and Activities of Daily Living of Patients With Poststroke Cognitive Impairment: A Systematic Review and Meta-Analysis. Arch. Phys. Med. Rehabil..

[88] De Giorgi R., Fortini A., Aghilarre F., Gentili F., Morone G., Antonucci G., Vetrano M., Tieri G., Iosa M. (2023). Virtual Art Therapy: Application of Michelangelo Effect to Neurorehabilitation of Patients with Stroke. J. Clin. Med..

[89] Castillo J.F.V., Vega M.F.M., Cardona J.E.M., Lopez D., Quiñones L., Gallo O.A.H., Lopez J.F. (2024). Design of Virtual Reality Exergames for Upper Limb Stroke Rehabilitation Following Iterative Design Methods: Usability Study. JMIR Serious Games.

[90] Pilacinski A., Pinto A., Oliveira S., Araújo E., Carvalho C., Silva P.A., Matias R., Menezes P., Sousa S. (2023). The Robot Eyes Don’t Have It. The Presence of Eyes on Collaborative Robots Yields Marginally Higher User Trust but Lower Performance. Heliyon.

[91] Park S., Lee G. (2020). Full-Immersion Virtual Reality: Adverse Effects Related to Static Balance. Neurosci. Lett..

[92] De Luca R., Manuli A., De Domenico C., Lo Voi E., Buda A., Maresca G., Bramanti A., Calabrò R.S. (2019). Improving Neuropsychiatric Symptoms Following Stroke Using Virtual Reality. Medicine.

[93] Calabrò R.S., Bonanno M., Torregrossa W., Cacciante L., Celesti A., Rifici C., Tonin P., De Luca R., Quartarone A. (2023). Benefits of Telerehabilitation for Patients With Severe Acquired Brain Injury: Promising Results From a Multicenter Randomized Controlled Trial Using Nonimmersive Virtual Reality. J. Med. Internet Res..

[94] Choukou M.-A., He E., Moslenko K. (2023). Feasibility of a Virtual-Reality-Enabled At-Home Telerehabilitation Program for Stroke Survivors: A Case Study. J. Pers. Med..

